# The impact of the COVID-19 pandemic on Sexually Transmitted Infections surveillance data: incidence drop or artefact?

**DOI:** 10.1186/s12889-021-11630-x

**Published:** 2021-09-07

**Authors:** Alexis Sentís, Albert Prats-Uribe, Evelin López-Corbeto, Marcos Montoro-Fernandez, Daniel Kwakye Nomah, Patrícia Garcia de Olalla, Lilas Mercuriali, Núria Borrell, Víctor Guadalupe-Fernández, Juliana Reyes-Urueña, Jordi Casabona, Catalan HIV and STI Surveillance Group, A. Sentís, E. López, V. Gonzalez, R. Lugo, M. P. Bonamusa, J. Reyes, J. Casabona, P. Garcia de Olalla, Lilas Mercuriali, R. Clos, R. Rodriguez, M. Masdeu, M. Ros, P. Simon, I. Avellaneda, A. Artigas, C. Rius, M. Company, M. Danés, N. Camps, R. M. Vileu, G. Ferrús, N. Borrell, S. Minguell, J. Ferràs, I. Parrón, I. Mòdol, A. Martinez, P. Godoy, M. A. Tarrès, J. Pérez, M. Boldú, I. Barrabeig, E. Donate, L. Clotet, M. R. Sala, M. Carol, V. Guadalupe-Fernández, J. Mendioroz, P. Ciruela, G. Carmona, R. Mansilla, J. L. Martínez, S. Hernández

**Affiliations:** 1 Centre of epidemiological studies on sexually transmitted infections and AIDS of Catalonia (CEEISCAT). Department of Health. Generalitat of Catalonia, Badalona, Spain; 2 Spanish Consortium for Research on Epidemiology and Public Health (CIBERESP), Instituto de Salud Carlos III, Madrid, Spain; 3 Pompeu Fabra University (UPF), Barcelona, Spain; 4 Epiconcept, Epidemiology Department, Paris, France; 5 Centre for Statistics in Medicine, Botnar Research Centre, NDORMS, University of Oxford, Oxford, UK; 6 Department of Paediatrics, Obstetrics and Gynecology and Preventive Medicine, Universitat Autónoma de Barcelona, Badalona, Spain; 7 Epidemiological Service of Public Health Agency of Barcelona, Barcelona, Spain; 8 Epidemiological Surveillance and Response to Public Health Emergencies Service in Tarragona Camp, Agency of Public Health of Catalonia, Generalitat of Catalonia, Tarragona, Spain; 9 Epidemiological Surveillance and Response to Public Health Emergencies Service in Central Catalonia, Agency of Public Health of Catalonia, Generalitat of Catalonia, Manresa, Spain

**Keywords:** Public health, Surveillance, communicable diseases, Sexually transmitted infections, Interrupted time series, COVID-19, Lockdown, Trends, Epidemiology

## Abstract

**Background:**

Before the COVID-19 pandemic, Sexually transmitted infections (STIs) were increasing in Europe, and Spain and Catalonia were not an exception. Catalonia has been one of the regions with the highest number of COVID-19 confirmed cases in Spain. The objective of this study was to estimate the magnitude of the decline, due to the COVID-19 pandemic, in the number of STI confirmed cases in Catalonia during the lockdown and de-escalation phases.

**Methods:**

Interrupted time series analysis was performed to estimate the magnitude of decline in the number of STI reported confirmed cases - chlamydia, gonorrhoea, syphilis, and lymphogranuloma venereum- in Catalonia since lockdown with historical data, from March 13th to August 1st 2020, comparing the observed with the expected values.

**Results:**

We found that since the start of COVID-19 pandemic the number of STI reported cases was 51% less than expected, reaching an average of 56% during lockdown (50% and 45% during de-escalation and new normality) with a maximum decrease of 72% for chlamydia and minimum of 22% for syphilis. Our results indicate that fewer STIs were reported in females, people living in more deprived areas, people with no previous STI episodes during the last three years, and in the HIV negative.

**Conclusions:**

The STI notification sharp decline was maintained almost five months after lockdown started, well into the new normality. This fact can hardly be explained without significant underdiagnosis and underreporting. There is an urgent need to strengthen STI/HIV diagnostic programs and services, as well as surveillance, as the pandemic could be concealing the real size of the already described re-emergence of STIs in most of the European countries.

**Supplementary Information:**

The online version contains supplementary material available at 10.1186/s12889-021-11630-x.

## Background

Before the coronavirus disease 2019 (COVID-19) pandemic, the number of new cases of mandatory notifiable sexually transmitted infections (STIs) was increasing in many European countries. Catalonia also had a pronounced rise of chlamydia, gonorrhoea, syphilis and lymphogranuloma venereum (LGV). For the last five years, Catalonia presented the highest incidence rates of Spain in all mandatory notifiable STIs, with a 20% annual increase. The rates were highest among males who have sex with males (MSM) and young adults, mostly females aged between 20 to 24 [1]. STIs represent one of the highest burdens of disease in adolescents and young females, leading to miscarriage, pelvic inflammatory disease, and increased risk to acquire human immunodeficiency virus (HIV) [2–4]. According to data published by the Government of Catalonia, the COVID-19 pandemic hit Catalonia harshly, with 676.863 confirmed cases and 22.124 deaths by May 24th, 2021, with one of the highest number of confirmed cases in Spain [5, 6]. On March 13, the Spanish government announced a countrywide lockdown, with a mandatory stay at home ordinance, with some exceptions, such as purchasing food or medicine, going to work or attending to emergencies. Visiting intimate partners were not included in the exceptions [7]. The combined effects of the lockdown and the unprecedented pressure on health systems might have reduced the capacity to detect STIs, potentially leading to increased transmission and more severe sequelae, or a decrease in the incidence because of less exposure. The objective of this study was to estimate the magnitude of the decline, due to the COVID-19 pandemic, in the number of STI reported confirmed cases in Catalonia during the lockdown and de-escalation phases comparing the observed and expected values.

## Methods

### The Catalan HIV/STI surveillance systems

We used epidemiological data from all STI confirmed cases reported to the Catalan HIV/STI Registry of Catalonia [8] through the Epidemiological Repository of Catalonia (REC), an electronic database that collects data reported from health care professionals and laboratories by means of standardized notification forms and epidemiological questionnaires, both electronically or in paper. According to the mandatory notification of diseases and outbreaks Catalan regulation (Health Department of Government of Catalonia article 13 of law 67/2010, 25 May 2010), nominal notification of syphilis, gonorrhoea, and LGV cases has been mandatory since 2007 and chlamydia since 2015; the notification of HIV cases was voluntary between 2001 and 2010 when it also became mandatory and nominal. The European Centre for Disease Prevention and Control (ECDC) guidelines are used for case definition criteria and all reported cases are reviewed by epidemiologists from the Epidemiological Surveillance Network of Catalonia (XVEC) to ensure completeness and validity of the data.

### Study variables

Sex, age group, and country of birth were collected from REC. Deprivation index (calculated by the Agency of Health Quality and Assessment of Catalonia) was based on the patient area of residence and categorized in quintiles, with the first quintile being the least deprived [9]. Multiple episodes by the same STI in the same individual were considered reinfections when evidence of it, proper treatment, and minimum length of time between reports existed. Time periods were defined according to specific STI treatment duration and follow up recommendations, being 364, 29, 119 and 119 days respectively for syphilis, gonorrhoea, chlamydia and LGV [10]. HIV status was confirmed checking the HIV status among individuals who had one single or more STI episodes during the study period within the Catalan HIV/STI Registry of Catalonia where previous and simultaneous HIV coinfections can be identified.

### Interrupted time series and data analysis

We analysed STI reported cases between August 1st 2017 and August 1st 2020 in Catalonia. We have included three years of follow-up not only in order to estimate the reported cases we would have had from lockdown - from March 13th to August 1st, 2020- but also to capture potential seasonal or cyclic changing patterns. For each of the mentioned variables, among the STI reported confirmed cases, the total number and its distribution in the respective categories were calculated, before lockdown, during lockdown (March 13th to April 27th), on de-escalation phases (April 28th until June 21st), and during the new normality phase (since June 22nd). The main objective of the de-escalation plan was to ensure maintaining the protection of public health while gradually recovering the common daily life and economic activities after the lockdown. This transition to a “new normality” was gradual, asymmetrical, and coordinated with the autonomous communities [11]. We used these dates as change points for an interrupted time series (ITS) analysis of daily STI reported cases (overall and separately for each of them). Reported cases were modelled as autoregressive integrated moving average (ARIMA) processes to estimate expected number of STI reported cases in each specific study period since lockdown based on pre-lockdown data. We calculated the overall drop in number of STI reported cases, to estimate the magnitude of the decline in STI reported cases with historical data comparing the observed and expected values.

## Results

When comparing with pre-lockdown period’s data, the STI reported cases per day decreased by almost 50% in all three COVID-19 related study periods: during lockdown, de-escalation and new normality periods (from 43.8 STI reported cases/day pre-lockdown to 22.2, 23.4 and 27.9 respectively). The proportion of syphilis and LGV slightly increased (from 15% and 1.7% pre-lockdown) among the overall STI reported cases in all three COVID-19 related study periods (respectively in the three study periods to 16.9%, 18%, and 17.3% for syphilis and 2.8%, 2.3% and 3.1% for LGV) meanwhile gonorrhoea and chlamydia had a small decrease (from 31.9% and 51.3% pre-lockdown to 30.3% and 49.1% in the “new normality” period for gonorrhoea and for chlamydia respectively). In addition, the proportion of STI reported cases from females was reduced when compared to males (approximately 5% between pre-lockdown and new normality periods). STI reported cases that came from areas with higher socioeconomic status increased by 5% over post-lockdown periods (see Table 1). The proportion of missing data for country of birth was high during all the different study periods and increased by 25% during lockdown (from 56.7% missing values in pre-lockdown data to 84% during lockdown). The proportion of STI reported cases in people coinfected by HIV, as well as the proportions of STI reported cases considered as reinfections, increased from pre-lockdown to new normality periods (5.2% to 6.1% and 11.7% to 22.2%, respectively). The proportion of reported cases in each age groups was similar when comparing the study periods with previous or historical data (see Table 1).

**Table 1:** Epidemiological characteristics of the STI reported confirmed cases during the different study periods^a^ in Catalonia, August 1st 2017 to August 1st 2020

	Total		Pre-lockdown		Lockdown		De-escalation Phases		New Normality	
Number of reported cases	*N* = 45,181		*N* = 41,802		*N* = 997		*N* = 1266		*N* = 1116	
Days,n°reported cases/days	1096	41.2	954	43.8	45	22.2	54	23.4	40	27.9
	N	%	N	%	N	%	N	%	N	%
**STI**										
Chlamydia	23,095	51.1	21,463	51.3	472	47.3	611	48.3	549	49.1
Gonorrhoea	14,406	31.9	13,340	31.9	329	33.0	398	31.4	339	30.3
LGV^b^	815	1.8	723	1.7	28	2.8	29	2.3	35	3.1
Syphilis	6865	15.2	6276	15.0	168	16.9	228	18.0	193	17.3
**Sex**										
Females	17,860	39.5	16,679	39.9	340	34.1	453	35.8	388	34.7
Males	27,321	60.5	25,123	60.1	657	65.9	813	64.2	728	65.2
**Age (mean, SD**^**c**^)	31.7	11.1	31.7	11.1	31.9	10.6	32.5	11.9	32.3	11.4
**Age group**										
< 20	5776	12.8	5384	12.9	113	11.3	154	12.2	125	11.2
20 to 29	17,334	38.4	16,052	38.4	376	37.7	477	37.7	429	38.4
30 to 39	12,076	26.7	11,180	26.7	282	28.3	321	25.4	293	26.3
40 to 49	6702	14.8	6177	14.8	155	15.5	198	15.6	172	15.4
50 to 59	2334	5.20	2124	5.1	56	5.6	83	6.6	71	6.4
≥ 60	959	2.1	885	2.1	15	1.5	33	2.6	26	2.3
**Deprivation index**										
First quintile	11,221	24.8	10,288	24.6	286	28.70	344	27.20	303	27.2
Second quintile	8133	18.0	7436	17.8	214	21.50	240	19.00	243	21.8
Third quintile	5329	11.8	4915	11.8	135	13.50	155	12.20	124	11.1
Fourth quintile	6150	13.6	5717	13.7	106	10.60	193	15.20	134	12.0
Fifth quintile	8246	18.3	7618	18.2	185	18.60	236	18.60	207	18.5
Missing	6102	13.5	5828	13.9	71	7.10	98	7.70	105	9.4
**Country of birth**										
Spain	13,861	30.7	13,316	31.9	136	13.6	207	16.4	202	18.1
Others	4913	10.9	4785	11.4	24	2.4	56	4.4	48	4.3
Missing	26,407	58.4	23,701	56.7	837	84.0	1003	79.2	866	77.6
**Reinfeccion**										
No	39,619	87.7	36,893	88.3	838	84.1	1019	80.5	869	77.9
Yes	5562	12.3	4909	11.7	159	15.9	247	19.5	247	22.1
**HIV status**										
Negative	42,809	94.8	39,628	94.8	937	94.0	1196	94.5	1048	93.9
Positive	2372	5.2	2174	5.2	60	6.0	70	5.5	68	6.1

^a^Pre-lockdown: from August 1st 2017 to March 12th 2020, lockdown: from March 13th 2020 to April 27^th^2020, de-escalation phases: from April 28th 2020 to June 21st 2020, new normality: from June ^22nd^ 2020 to August 1st 2020;

^b^LGV: Lymphogranuloma venereum;

^c^SD: Standard deviation

**Fig. 1:**
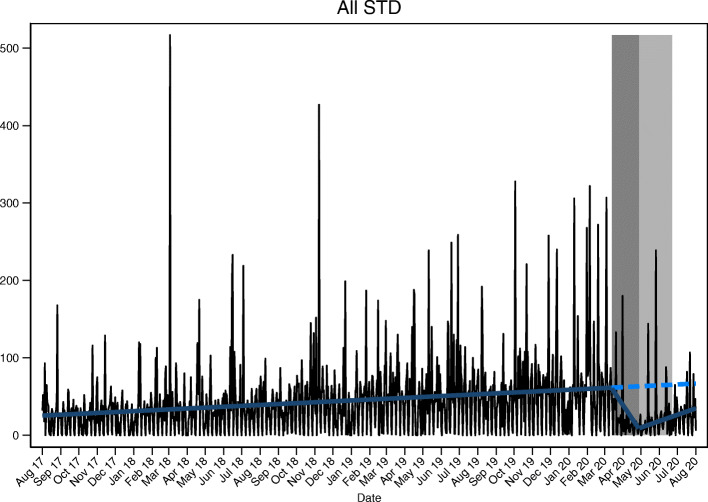
Observed and expected time series trends of daily STI reported confirmed cases in Catalonia during the COVID-19 pandemic, August 1st 2017 - August 1st 2020 (dark grey: lockdown, light grey: de-escalation phases)

In the ITS (see Table 2, Fig. 1, and supplemental material, figure S1–S4), we observed how the number of all STI reported cases were only 49% of the expected number (decrease of − 51%, confidence interval (CI): − 59% to − 38%), based on pre-lockdown data (since August 1st 2020), with variations in the different study periods; being only 44% of the expected reported cases during lockdown (decrease of − 56%, CI: − 63% to − 46%) and slowly increasing to 55% of the expected since the new normality began on June 22nd (decrease of − 45%, CI: − 54% to − 30%). When analysing the results by type of infection we found that chlamydia’s reported cases which represent more than 50% of all STI reported cases, had the highest decrease in notification over post-lockdown periods, with observed reported cases reaching only 28% of the expected values. Conversely, we observed that the decrease of syphilis reported cases was lower, with observed reported cases reaching 78% of those expected (see Table 2 and supplemental material, figure S1 and figure S3).

**Table 2:** Comparing observed and expected number of STI reported confirmed cases during the different study periods^a^ in Catalonia, August 1st 2017 to August 1st 2020

	Periods	observed	expected	upper CI^b^	lower CI^b^	difference		upper CI^b^		lower CI^b^	
**All STIs**	**Pre-lockdown**	41,814									
	**Lockdown**	997	2264	2681	1846	− 1267	−56%	− 1684	−63%	− 849	−46%
	**De-escalation**	1266	2546	3078	2015	− 1280	−50%	− 1812	−59%	− 749	−37%
	**New Normality**	1116	2026	2446	1606	− 910	−45%	− 1330	−54%	− 490	−30%
	**Total**	3379	6836	8205	5467	− 3457	−51%	− 4826	−59%	− 2088	−38%
**Chlamydia**	**Pre-lockdown**	21,463									
	**Lockdown**	472	1607	1731	1483	−1135	−71%	− 1259	−73%	− 1011	−68%
	**De-escalation**	611	2376	2535	2216	− 1765	−74%	− 1924	−76%	− 1605	−72%
	**New Normality**	549	1870	1997	1743	− 1321	−71%	− 1448	−73%	− 1194	−68%
	**Total**	1632	5853	6264	5442	− 4221	−72%	− 4632	−74%	− 3810	−70%
**Gonorrhoea**	**Pre-lockdown**	13,340									
	**Lockdown**	329	747	889	605	−418	−56%	−560	−63%	−276	−46%
	**De-escalation**	398	857	1037	678	− 459	−54%	− 639	−62%	−280	−41%
	**New Normality**	339	670	812	529	− 331	−49%	− 473	− 58%	−190	−36%
	**Total**	1066	2275	2738	1812	− 1209	−53%	− 1672	−61%	− 746	− 41%
**Syphilis**	**Pre-lockdown**	6276									
	**Lockdown**	168	250	337	164	−82	−33%	− 169	−50%	4	2%
	**De-escalation**	228	284	392	177	−56	−20%	− 164	−42%	51	29%
	**New Normality**	193	218	302	134	−25	−11%	−109	−36%	59	44%
	**Total**	589	753	1030	475	−164	−22%	− 441	−43%	114	24%
**LGV**^c^	**Pre-lockdown**	723									
	**Lockdown**	28	54	62	47	−26	−48%	−34	−55%	−19	−40%
	**De-escalation**	29	80	89	71	−51	−64%	−60	−67%	−42	−59%
	**New Normality**	35	56	63	49	−21	−38%	−28	−45%	−14	−29%
	**Total**	92	191	214	167	−99	−52%	− 122	−57%	−75	−45%

^a^Pre-lockdown: from August 1st 2017 to March 12th 2020, lockdown: from March 13th 2020 to April 27^th^2020, de-escalation phases: from April 28th 2020 to June 21st 2020, new normality: from June ^22nd^ 2020 to August 1st 2020; bCI, confidence interval; cLGV, lymphogranuloma venereum

## Discussion

We found that since the start of COVID-19 pandemic the number of STI reported cases was 51% less than expected, reaching an average of 56% during lockdown (50 and 45% during de-escalation and new normality) with a maximum decrease of 72% for chlamydia and minimum of 22% for syphilis. Our results indicate that STIs were less reported in females, people living in more deprived areas, people with no previous STI episodes during the last three years, and without HIV infection.

We hypothesize that the decline in the number of STI reported cases was higher in females based on the annual STI notification rates in the region where chlamydia has been usually higher among females and syphilis among males [1]. STIs, including chlamydia infection are predominantly asymptomatic in females, and are detected primarily through screening. During lockdown, mobility restrictions may have decreased healthcare seeking behaviour in asymptomatic individuals with high-risk exposures. This decrease could be even greater for people living in more deprived areas. Additionally, people who already visited sexual health care for previous STI episodes in the last three years, or HIV positive are more likely to seek health care.

To the best of our knowledge, few analysis have described the estimated magnitude and impact of the COVID-19 pandemic on the expected number of STIs compared to the most recent historical data. Although few articles have analysed STI incidences during lockdown, some authors argue that the plausible decrease of sexually relations during the COVID-19 pandemic may partially explain the apparent drop in the number of STI incidence [12–14]. In spite of these, different stakeholders have started raising awareness about disruptions in sexual health services including STI and HIV testing and detection [15, 16]. Moreover, it has been estimated that in the Atlanta (State of Georgia, United States), if sexual behaviour rebounds while service interruption persists, cases will increase in hundreds for HIV and in thousands for STIs for the next five years [17]. Berzkalns et al. performed a study in King County (State of Washington, United States) where the number of sexual health clinic visits decreased 55% during lockdown. Although after lockdown numbers returned to pre-lockdown values, around lockdown, from January–July 2020, the number of STIs diagnosed declined differently depending on the STI, from 9% for gonorrhoea to 22% for early latent syphilis [18]. They suggested that a real decline may have happened, but the larger decline in asymptomatic STIs probably indicates decreased screening. Similarly, Chow et al. described that, although a relevant decrease in the total number of consultations occurred in the Melbourne Sexual Health Centre during the lockdown, for more severe conditions such as pelvic inflammatory disease or infectious syphilis, a similar number of consultations to the pre-lockdown period was observed [19]. Recently, an article from the EuroTEST COVID-19 impact assessment consortium described that, among 34 countries in the World Health Organization (WHO) European Region and in different testing settings, 95% of them declared to have tested less than half the expected number of people between March and May 2020, a decline that continued at lesser degrees until August 2020 [20]. Then, this decline probably is due to the effect of a combination of factors; changes in the people’s behaviour, sexual relationships or fear of visiting a health care setting [21], less available resources to diagnose and notify STIs (including human resources), and surveillance systems which were not able to integrate the immediate reaction to a pandemic, while coping with their regular surveillance activities.

## Conclusions

Our results showed that the STI notification sharp decline was maintained almost five months since lockdown, well into the new normality. This can hardly be explained without significant underdiagnoses and underreporting. The gradual increase in the number of STI reported cases that we observed after lockdown may be pointing out the possibility that lockdowns did not completely disrupt STI transmission. As discussed in the present article, with the available current scientific evidence, it seems that the observed decrease in the number of STI reported cases during the current COVID-19 pandemic is probably due to a combination of factors. More research is needed in order to disentangle the specific role and relevance that has had underdiagnosis, underreporting, and the decrease in sexual risk activities and other potential factors in this decline. Finally, we truly believe that there is an urgent need to strengthen STI/HIV diagnostic programs and services, as well as surveillance, as the pandemic could be concealing the real size of the already described re-emergence of STIs [22].

## Supplementary Information

**Additional file 1: Fig. S1**. Expected and observed time series trends of daily Chlamydia reported confirmed cases in Catalonia, August 1st 2017 - August 1st 2020 (dark grey: lockdown, light grey: de-escalation phases). **Fig. S2**. Expected and observed time series trends of daily Gonorrhoea reported confirmed cases in Catalonia, August 1st 2017 - August 1st 2020 (dark grey: lockdown, light grey: de-escalation phases). **Fig. S3**. Expected and observed time series trends of daily Syphilis reported confirmed cases in Catalonia, August 1st 2017 - August 1st 2020 (dark grey: lockdown, light grey: de-escalation phases). **Fig. S4**. Expected and observed time series trends of daily lymphogranuloma venerum (LGV) reported cases in Catalonia, August 1st 2017 - August 1st 2020 (dark grey: lockdown, light grey: de-escalation phases).

## Data Availability

Public access to the database(s) is close. Data sharing is not possible because patients’ individual privacy could be compromised. Although data was de-identified to be handled, some aggregated results could be sensitive when communicated at a population level. For this reason, the analysis and dissemination of this data is handled by public health authorities with surveillance responsibilities and avoiding disaggregation by small geographical areas.

## Abbreviations

COVID-19: The coronavirus disease 2019
STI: Sexually transmitted infections
LGV: Lymphogranuloma venereum
MSM: Males who have sex with males
HIV: Human immunodeficiency virus
REC: Epidemiological repository of Catalonia
XVEC: The Epidemiological Surveillance Network of Catalonia
ECDC: European Centre for Disease Prevention and Control
ITS: Interrupted time series
ARIMA: Autoregressive integrated moving average
CI: Confidence interval
WHO: World Health Organization

## References

[1] Centre of epidemiological studies on sexually transmitted infections and AIDS of Catalonia (CEEISCAT). Vigilància epidemiològica de les infeccions de transmissió sexual a Catalunya. 2019. www.ceeiscat.cat. Accessed 7 Aug 2020.

[2] STD Facts - STDs & Pregnancy Detailed Fact Sheet. https://www.cdc.gov/std/pregnancy/stdfact-pregnancy-detailed.htm. Accessed 24 May 2021.

[3] WHO. Sexually transmitted infections (STIs). https://www.who.int/news-room/fact-sheets/detail/sexually-transmitted-infections-(stis). Accessed 7 Aug 2020.

[4] HIV/AIDS & STDs. https://www.cdc.gov/std/hiv/default.htm. Accessed 22 Feb 2021.

[5] Situación de COVID-19 en España a 19 de mayo de 2021. Equipo COVID-19. RENAVE. CNE. CNM (ISCIII). Informe n^o^ 79. https://www.isciii.es/QueHacemos/Servicios/VigilanciaSaludPublicaRENAVE/EnfermedadesTransmisibles/Documents/INFORMES/Informes COVID-19/INFORMES COVID-19 2021/Informe n^o^ 79. Situación de COVID-19 en España a 19 de mayo de 2021.pdf. Accessed 24 May 2021.

[6] Generalitat de Catalunya, Departament de Salut. Dades COVID https://dadescovid.cat/. Accessed 24 May 2021.

[7] Real Decreto 463/2020, de 14 de marzo, por el que se declara el estado de alarma para la gestión de la situación de crisis sanitaria ocasionada por el COVID-19. Boletin oficial del estado. Sec. I. Pág. 25390. https://www.boe.es. Accessed 24 May 2021.

[8] SIVES 2015 - Sistema Integrat de Vigilància Epidemiològica de la SIDA/VIH/ITS a Catalunya. 2015. https://scientiasalut.gencat.cat/bitstream/handle/11351/3418/informe_SIVES_2015_informe_epidemiologic_CEEISCAT_2015.pdf.pdf?sequence=1&isAllowed=y. Accessed 27 Mar 2021.

[9] Agency for Health Quality and Assessment of Catalonia. Nou indicador socioeconòmic per al finançament de les ABS. Observatori del Sistema de Salut de Catalunya. 2017. http://observatorisalut.gencat.cat/ca/observatori-desigualtats-salut/indicador_socioeconomic_2015/. Accessed 6 Aug 2020.

[10] Centers for Disease Control and Prevention. Sexually Transmitted Diseases Treatment Guidelines, 2015. MMWR Recomm Rep 2015; 64 (No. RR-3): 33, 37, 57, 64.https://www.cdc.gov/std/tg2015/toc.htm. Accessed 2 Sep 2020. PMC5885289 26042815

[11] Plan para la transición hacia una nueva normalidad. 28 de abril 2020. Gobierno de España. Ministerio de Sanidad. https://www.lamoncloa.gob.es/consejodeministros/resumenes/Documents/2020/PlanTransicionNuevaNormalidad.pdf. Accessed 25 May 2021.

[12] Mohamed A, Hammoud LM, et al. Physical distancing due to COVID-19 disrupts sexual behaviours among gay and bisexual men in Australia. JAIDS J Acquir Immune Defic Syndr. https://pubmed.ncbi.nlm.nih.gov/32740374/. 10.1097/QAI.0000000000002462 32740374

[13] Alpalhão M, Filipe P (2020). The impacts of isolation measures against SARS-CoV-2 infection on sexual health. AIDS Behav.

[14] Balestri R, Magnano M, Rizzoli L, Infusino SD, Urbani F, Rech G. STIs and the COVID-19 pandemic: the lockdown does not stop sexual infections. J Eur Acad Dermatol Venereol. 2020:jdv.16808. 10.1111/jdv.16808. 10.1111/jdv.16808 PMC7405161 32652791

[15] Tang K, Gaoshan J, Ahonsi B (2020). Sexual and reproductive health (SRH): a key issue in the emergency response to the coronavirus disease (COVID-19) outbreak. Reprod Health.

[16] Sanchez TH, Zlotorzynska M, Rai M, Baral SD (2020). Characterizing the impact of COVID-19 on men who have sex with men across the United States in April, 2020. AIDS Behav.

[17] Jenness SM, Le Guillou A, Chandra C, Mann LM, Sanchez T, Westreich D, et al. Projected HIV and bacterial STI incidence following COVID-related sexual distancing and clinical service interruption. medRxiv. 2020. 10.1101/2020.09.30.20204529. 10.1093/infdis/jiab051 PMC7928867 33507308

[18] Berzkalns A, Thibault CS, Barbee LA, Golden MR, Khosropour C, Kerani RP (2021). Decreases in reported sexually transmitted infections during the time of COVID-19 in King County, WA: decreased transmission or screening?. Sex Transm Dis.

[19] Chow EPF, Hocking JS, Ong JJ, Phillips TR, Fairley CK. Sexually transmitted infection diagnoses and access to a sexual health service before and after the National Lockdown for COVID-19 in Melbourne, Australia. Open Forum Infect Dis. 2021;8(1). 10.1093/ofid/ofaa536. 10.1093/ofid/ofaa536 PMC7665697 33506064

[20] Simões D, Stengaard AR, Combs L, Raben D (2020). Impact of the COVID-19 pandemic on testing services for HIV, viral hepatitis and sexually transmitted infections in the WHO european region, march to august 2020. Eurosurveillance..

[21] Alessandra L, Francesca M, Maria Gabriella D, Massimo G, Antonio C, Mauro Z. Is COVID-19 affecting the epidemiology of STIs? The experience of syphilis in Rome. Sex Transm Infect. 2020; sextrans-2020-054543. 10.1136/sextrans-2020-054543. 10.1136/sextrans-2020-054543 32719105

[22] Williamson DA, Chen MY (2020). Emerging and reemerging sexually transmitted infections. N Engl J Med.

