# Supplementary material for: The impact of the COVID-19 pandemic on Sexually Transmitted Infections surveillance data: incidence drop or artefact?
Source: BMC Public Health. 2021 Sep 7;21:1637. doi: 10.1186/s12889-021-11630-x (PMC8423330; doi:10.1186/s12889-021-11630-x)
Supplement: Supplementary file 1 — Additional file 1: Fig. S1. Expected and observed time series trends of daily Chlamydia reported confirmed cases in Catalonia, August 1st 2017 - August 1st 2020 (dark grey: lockdown, light grey: de-escalation phases). Fig. S2. Expected and observed time series trends of daily Gonorrhoea reported confirmed cases in Catalonia, August 1st 2017 - August 1st 2020 (dark grey: lockdown, light grey: de-escalation phases). Fig. S3. Expected and observed time series trends of daily Syphilis reported confirmed cases in Catalonia, August 1st 2017 - August 1st 2020 (dark grey: lockdown, light grey: de-escalation phases). Fig. S4. Expected and observed time series trends of daily lymphogranuloma venerum (LGV) reported cases in Catalonia, August 1st 2017 - August 1st 2020 (dark grey: lockdown, light grey: de-escalation phases). [file 12889_2021_11630_MOESM1_ESM.docx]

**Supplemental material**

**Figure S1. Expected and observed time series trends of daily Chlamydia reported cases in Catalonia, August 1^st^ 2017 - August 1^st^ 2020** (dark grey: lockdown, light grey: de-escalation phases).

**
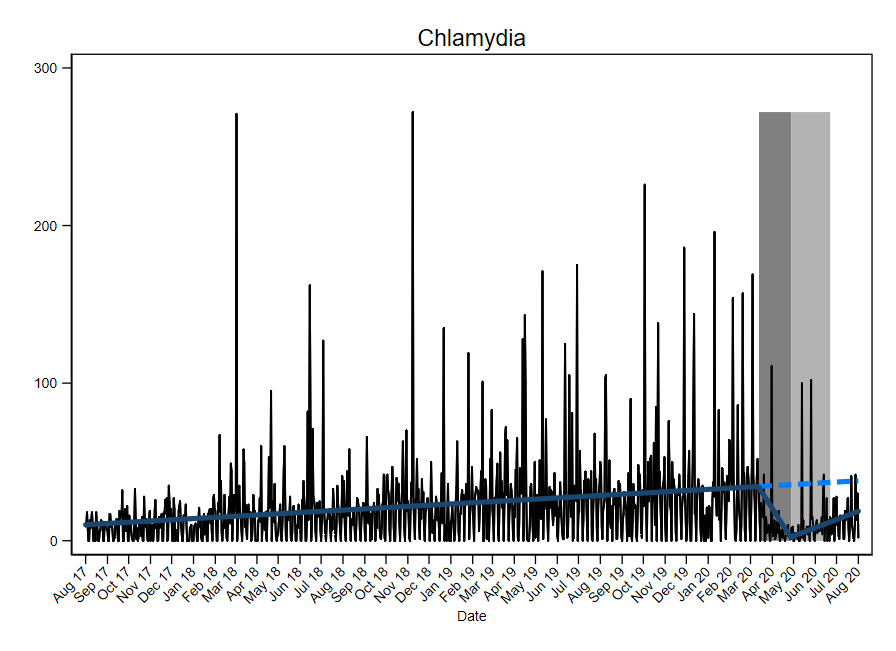
**

**Figure S2. Expected and observed time series trends of daily Gonorrhoea reported cases in Catalonia, August 1^st^ 2017 - August 1^st^ 2020** (dark grey: lockdown, light grey: de-escalation phases).

**
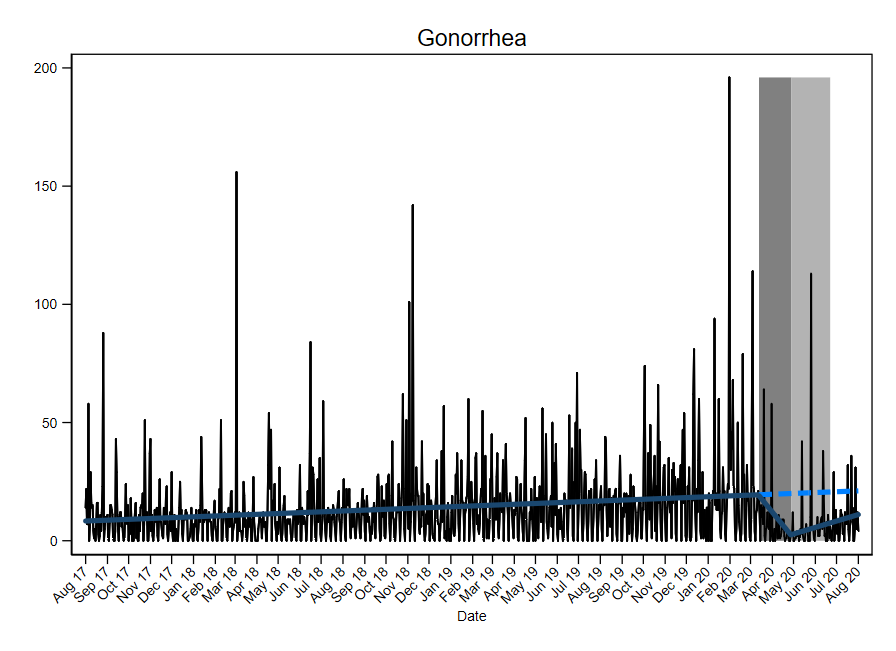
**

**Figure S3. Expected and observed time series trends of daily Syphilis reported cases in Catalonia, August 1^st^ 2017 - August 1^st^ 2020** (dark grey: lockdown, light grey: de-escalation phases).

**
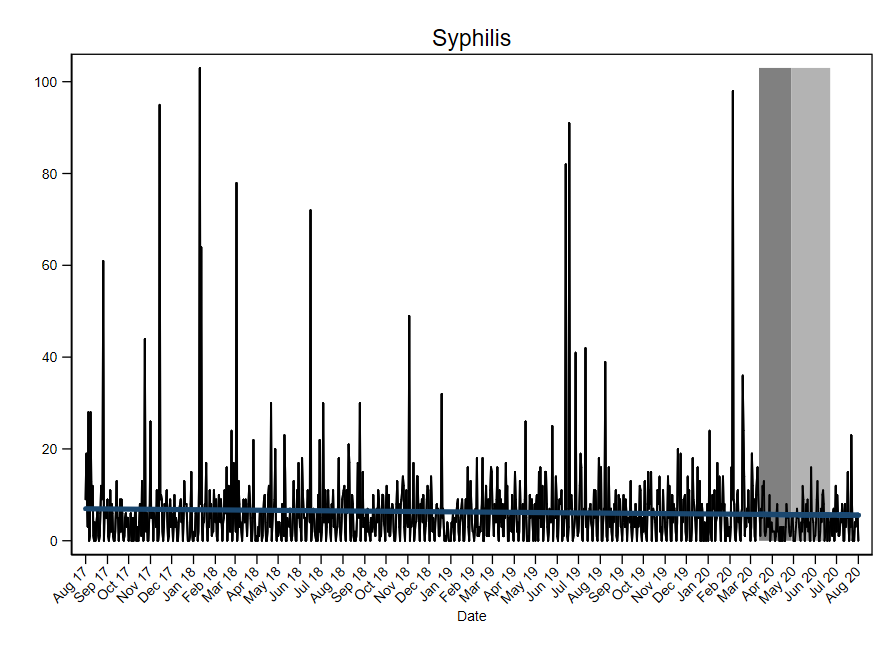
**

**Figure S4. Expected and observed time series trends of daily venereal lymphogranuloma (LGV) reported cases in Catalonia, August 1^st^ 2017 - August 1^st^ 2020** (dark grey: lockdown, light grey: de-escalation phases).

**
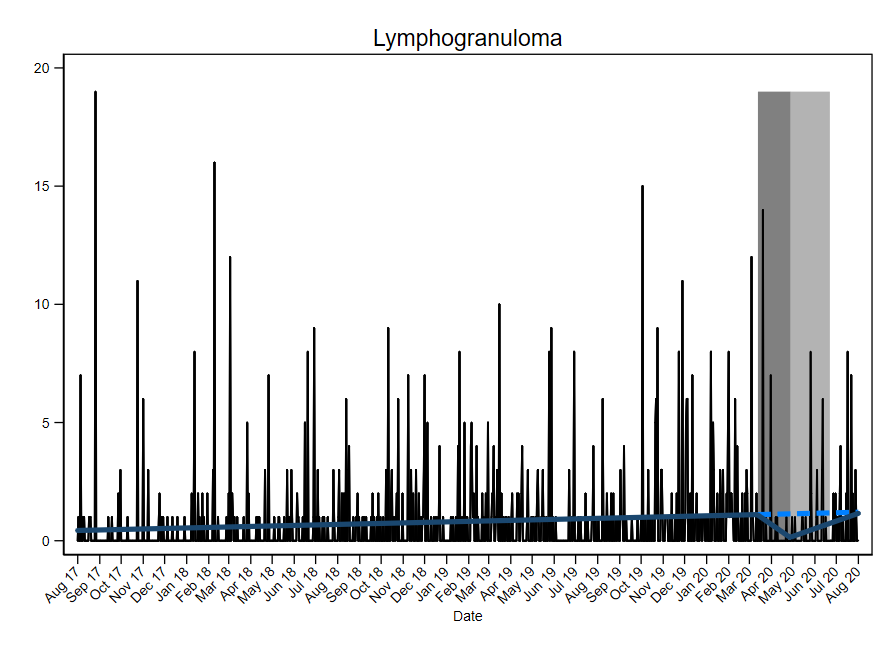
**
